# Simulating Highly Activated Sticking of H_2_ on Al(110): Quantum versus Quasi-Classical Dynamics

**DOI:** 10.1021/acs.jpcc.3c00426

**Published:** 2023-03-14

**Authors:** Theophile Tchakoua, Andrew D. Powell, Nick Gerrits, Mark F. Somers, Katharina Doblhoff-Dier, Heriberto F. Busnengo, Geert-Jan Kroes

**Affiliations:** † Leiden Institute of Chemistry, Gorlaeus Laboratories, Leiden University, P.O. Box 9502, 2300 RA Leiden, The Netherlands; ‡ Instituto de Física Rosario (IFIR), CONICET-UNR, Bv. 27 de Febrero 210 bis, 2000 Rosario, Argentina; § Facultad de Ciencias Exactas, Ingeniería y Agrimensura, UNR, Av. Pellegrini 250, 2000 Rosario, Argentina

## Abstract

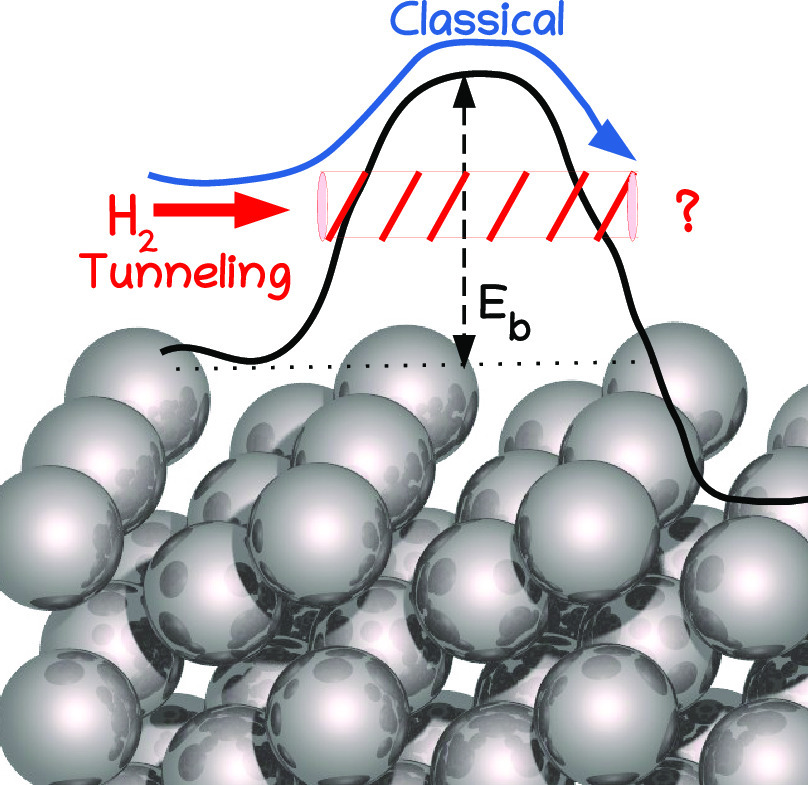

We evaluate the importance of quantum effects on the sticking of H_2_ on Al(110) for conditions that are close to those of molecular beam experiments that have been done on this system. Calculations with the quasi-classical trajectory (QCT) method and with quantum dynamics (QD) are performed using a model in which only motion in the six molecular degrees of freedom is allowed. The potential energy surface used has a minimum barrier height close to the value recently obtained with the quantum Monte Carlo method. Monte Carlo averaging over the initial rovibrational states allowed the QD calculations to be done with an order of magnitude smaller computational expense. The sticking probability curve computed with QD is shifted to lower energies relative to the QCT curve by 0.21 to 0.05 kcal/mol, with the highest shift obtained for the lowest incidence energy. Quantum effects are therefore expected to play a small role in calculations that would evaluate the accuracy of electronic structure methods for determining the minimum barrier height to dissociative chemisorption for H_2_ + Al(110) on the basis of the standard procedure for comparing results of theory with molecular beam experiments.

## 1 Introduction

The dissociative chemisorption (DC) of molecules on metal surfaces is of high practical interest, as the transition state (TS) of the DC reaction is often a rate-limiting state in overall heterogeneously catalyzed processes^1,2^ (such as ammonia production^3^ and steam reforming^4^), and most chemicals are made through heterogeneous catalysis.^5^ It is therefore important to be able to compute accurate barriers for DC on metals with electronic structure methods and to test the ability of density functional theory (DFT) to compute such barriers accurately. With more than 30,000 papers published annually,^6^ DFT is probably the most important electronic structure method applied to complex systems. While DFT has been tested extensively on databases of gas-phase reaction barriers,^7−10^ tests^11−13^ on databases of barrier heights for DC on metals^11,12^ are still scarce.

Unfortunately, reaction barrier heights are not observables.^14^ The way to validate the capability of electronic structure methods to accurately compute barrier heights is therefore to compute an observable that strongly depends on the barrier height.^14^ For DC on metals, this is the sticking probability (*S*_0_), which can be measured in a supersonic molecular beam experiment,^14,15^ as can be argued on the basis of the hole-model.^16^ The validation procedure therefore also requires dynamics calculations to be performed with an appropriate dynamical model and dynamical method.^14^ In this procedure, the electronic structure method is used to generate the forces acting on the atoms (either directly in ab initio molecular dynamics or density functional molecular dynamics calculations or indirectly from a potential energy surface that was fitted to ab initio data).^14^ If such calculations yield a *S*_0_ curve that is in good agreement with a high-quality experiment, and if the dynamical model and method used were of high enough accuracy, the minimum barrier height computed with the electronic structure method should be an accurate value of the TS energy, also allowing its use for benchmarking purposes.^11,12,14^

For DC of H_2_ on a metal surface, with few exceptions^17^ experiments measure *S*_0_, or effectively the DC probability averaged over the velocity distribution of and the rovibrational states populated in the molecular beam at the nozzle temperature (*T*_N_) used.^18−27^ With H_2_ being the lightest molecule, one might think that the sticking in such experiments should be highly influenced by quantum effects like tunneling and that this should be especially true if the barrier to DC is high. However, this is not necessarily true. For instance, on the basis of experiments on DC of H_2_ on Cu(111), it has been argued that at low incidence energies (*E*_i_), the reaction is dominated by vibrationally excited H_2_ in its *v* = 1 or even its *v* = 2 state, where *v* is the vibrational quantum number.^21^ With averaging over the rovibrational states, the question then becomes: is the sticking dominated by ″classical″, i.e., ″over the barrier″ reaction of H_2_ in highly excited vibrational and/or rotational states, or are quantum effects like tunneling highly important because most molecules that react are in low vibrational and rotational states with high Boltzmann populations, and their reaction is dominated by tunneling? In other words, to compute *S*_0_, does the quasi-classical trajectory (QCT) method^28,29^ suffice, or should one use a quantum dynamical (QD) method, like the time-dependent wave packet (TDWP) method?^30,31^ So far, existing evidence for H_2_ reacting on Cu(111)^32^ and Cu(211)^33^ suggests that quantum effects are not of large importance for *S*_0_ down to 0.01 or even to 0.001. Evidence concerning DC of H_2_ or D_2_ in specific single initial rovibrational states in some cases does suggest substantial differences between quantum and quasi-classical dynamics calculations,^34−40^ but as already indicated most experimental results for DC of H_2_ on metals represent averages over several rovibrational states. Additionally, in molecular beam experiments, the importance of quantum effects may depend on how wide the translational energy distributions of the beams are, as molecules in the high-energy tail of a beam might react more readily through a classical mechanism.

Here, the question we raised above (how important are quantum effects on the sticking of H_2_ on metal surfaces) is addressed for the DC of H_2_ on Al(110). There are several reasons for addressing this system. First, this system is representative of H_2_-metal DC reactions with a very high minimum barrier (i.e., >1 eV),^41^ as also found in, e.g., H_2_ + Ag(111)^32,42,43^ and H_2_ + Au(111).^32,43,44^ Second, this reaction has been investigated in experiments^20,45^ for which the velocity distributions used can be derived from actual time-of-flight (TOF) distributions and other experimental information that has been published.^45^ The information on these beams has been used successfully to accurately model experiments^18−20^ on the sticking of H_2_ and D_2_ on Cu(111),^46,47^ Cu(100),^47,48^ and Cu(110)^47^ that were performed with these beams. Finally, the H_2_ + Al(110) system is currently being used to investigate the performance of a new first-principles-based version of the specific reaction parameter (SRP) approach to DFT (SRP-DFT) in quasi-classical dynamics calculations. For the actual comparison with experiment that we intend to publish shortly (Powell et al., to be published), it will be important to know the importance of quantum effects, which are the focus of this study. Comparison with experiments is not yet the aim here, as this would also require inclusion of surface atom motion and electron–hole pair (ehp) excitation, which is beyond the scope of the present paper. In view of the usual way of validating an electronic structure method for barrier heights of DC (i.e., by computing the energy shift between a computed and a measured sticking probability curve^14,46^), the central question we will address is: To what extent may quantum effects be expected to shift the computed sticking probability curve for H_2_ + Al(110) along the incidence energy axis? While we address this question for H_2_ + Al(110), our results may also be relevant to the modeling of existing experiments on DC of H_2_ on Ag(111),^24^ or sticking experiments yet to be performed for H_2_ + Au(111).

Our paper is organized as follows: First, we describe the theoretical methods used in this work in Section 2. Section 2.1 describes the dynamical model and Section 2.2 the DFT method used to generate the electronic structure data describing the molecule–surface interaction. The corrugation reducing procedure^49^ used to interpolate the DFT data to generate a global PES is described in Section 2.3. Section 2.4 describes how we compute *S*_0_, the observable obtained in hyperthermal molecular beam experiments. The QD and the QCT methods that are used to obtain *S*_0_ for H_2_ + Al(110) are described in Sections 2.5.1 and 2.5.2, respectively. In Section 3, the results of the calculations are shown and discussed. Section 3.1 describes the computed PES, and Section 3.2 presents the *S*_0_ computed with QD and with the QCT method and their comparison. In Section 3.3, an attempt is made to underpin the size of the quantum effects predicted with an analysis of the QCT results and the characteristics of the molecular beams we simulate. Conclusions are provided in Section 4.

## 2 Method

### 2.1 Dynamical Model

**Figure 1:**
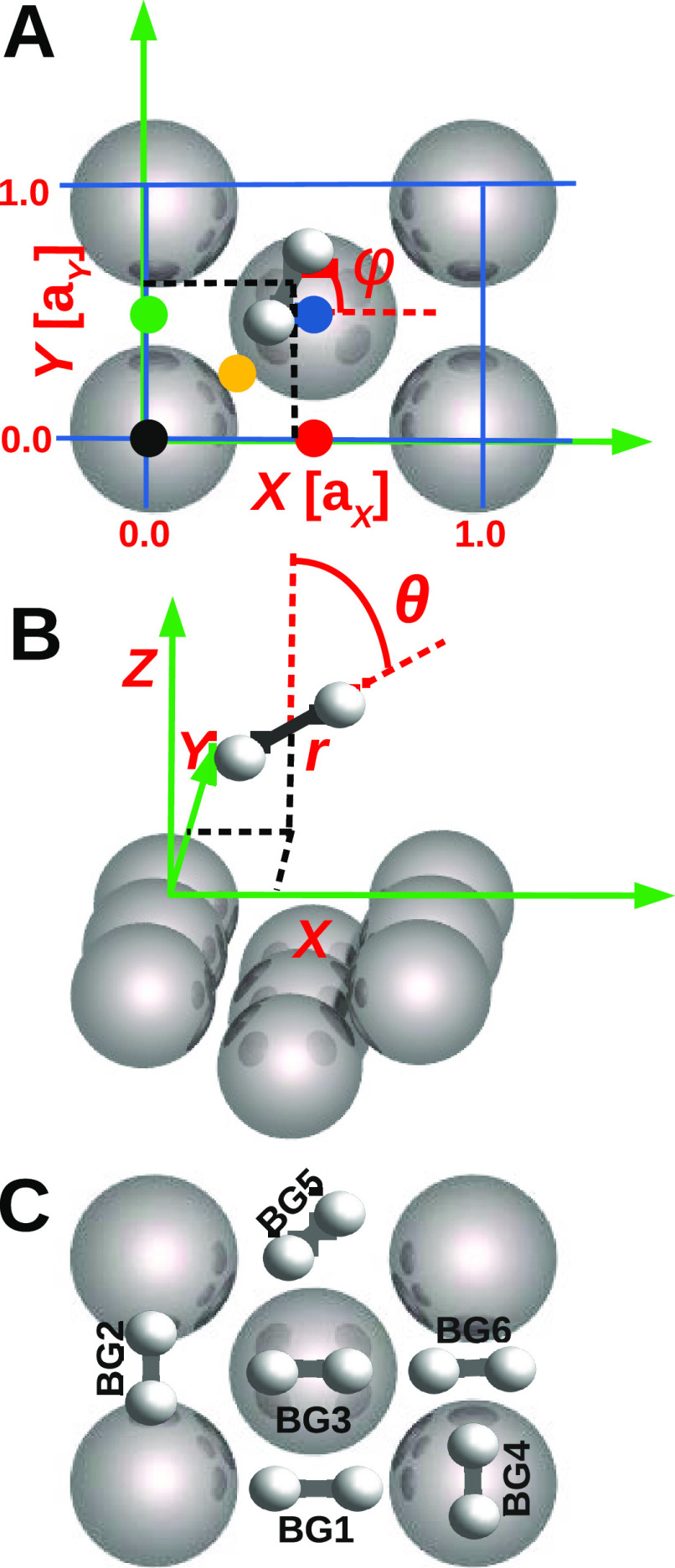
Top view (A) and side view (B) of the surface unit cell of Al(110), illustrating the six coordinates describing the geometry of the H_2_-Al(110) system in the BOSS model, and (C) six barrier geometries BG1-BG6. In (A), the black, green, red, blue, and yellow solid circles denote the top, short-bridge, long-bridge, hollow, and C-site, respectively.

In all calculations (in the QD and in the QCT calculations), the Born–Oppenheimer static surface (BOSS) model^14^ has been used. Within this model, the surface atoms are kept fixed in their ideal lattice positions and ehp excitation is neglected. Only the motion in the six H_2_ degrees of freedom (6D) is taken into account. Specifically, the molecular coordinates *X*, *Y*, and *Z* describe the motion of the molecule’s center of mass, where *Z* is the molecule–surface distance and *X* and *Y* describe the lateral positions (see Figure 1A,B). Furthermore, the H–H bond distance is given by *r* and the angular orientation of H_2_ by the polar angle θ the H_2_ bond makes with the surface normal and the azimuthal angle φ that the projection of the molecule’s bond axis on the surface makes with the *X*-axis (see Figure 1A,B). Figure 1A also shows the Al(110) surface unit cell and the high symmetry impact sites top, long-bridge, short-bridge, hollow, and the site we call the C-site.

As discussed below, the slab we used to model the Al surface mimics an ideal surface at a surface temperature (*T*_s_) of 220 K. We note that, with the way the slab has been set up for 220 K, we only include the effects of thermal expansion.^50^ Presently, we exclude the effect of the additional corrugation that surface atom motion can introduce in a real surface at 220 K and the effect of energy transfer between the molecule and the surface atoms.^50−53^ QCT calculations using the static corrugation model on the activated DC of H_2_ and D_2_ on Cu(111) at *T*_s_ = 120 K found little effect of the mentioned additional corrugation for *S*_0_ values as low as 10^–3^ (see Figure 13 of ref (51)). Likewise, density functional molecular dynamics (DFMD) calculations and QCT calculations investigating DC of D_2_ on Cu(111) found no detectable effect (within the statistical accuracy of the DFMD calculations) of the mentioned additional corrugation and of energy transfer at *T*_s_ = 120 K for *S*_0_ ≥ 10^–2^ (see Figure S1 of ref (53)). Given that DC of H_2_ on Al(110) is associated with even lower reaction probabilities,^20^ that the measurements on this system were performed at a somewhat higher *T*_s_ (220 K), and that the mass ratio between H and Al should be more conducive to energy transfer according to the Baule model^54^ than that between H and Cu, these effects might become more important for the system under investigation here. We believe however that for the current comparison between QD and QCT for the molecular beams of H_2_ that we simulate, these effects are not so relevant, although at present this is based on speculation and the answer may depend on whether the thermal motion may promote reactivity through tunneling by modulating the barrier height to DC.^55,56^ To our knowledge, work on how surface atom motion might affect the tunneling contribution to DC of H_2_ has not yet been performed. However, as stated previously, the effect of phonons will be considered in future work with quasi-classical dynamics (A.D. Powell et al., to be published).

### 2.2 DFT Method

Calculations of the H_2_-Al(110) molecule–surface interaction were performed using Kohn–Sham DFT.^57,58^ The density functional (DF) *E*_*xc*_^SRP71 – vdW2^used can be written as
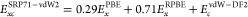
1

It contains 29% PBE^59^ exchange and 71% RPBE^60^ exchange, while the correlation part of the exchange-correlation functional was taken as the Rutgers–Chalmers vdW-DF2 correlation functional.^61^ As will be described in detail elsewhere (A.D. Powell et al., to be published), with this DF, an accurate fit is obtained of the barrier heights computed with diffusion Monte Carlo (DMC) for six barrier geometries of H_2_ + Al(110).^41^ For example, with the DF of eq 1 a TS energy of 25.4 kcal/mol is obtained, which is in good agreement with the DMC value of 25.1 kcal/mol.^41^ More details of the comparison with DMC data will be provided elsewhere.

In the plane wave DFT calculations, the Al(110) surface has been represented using a 10-layer thick Al slab. Details of how the slab was set up and adjusted to represent an Al(110) surface in which the atoms occupy the ideal lattice positions at 220 K are presented in Sections S1 and S3 of the Supporting Information (SI). A 3 × 3 surface unit cell was used, leading to a total of 90 Al atoms. A vacuum distance of 16.0 Å was used to separate the slab from its first periodic images in the supercell approach employed. The core electrons have been treated using pseudo-potentials within the projector augmented wave method^62,63^ (details are also presented in the Supporting Information). The energy cutoff for the plane wave expansion was 540 eV. The Brillouin zone has been sampled with an 8 × 8 × 1 Γ-centered grid of *k*-points. Convergence was facilitated using first-order Methfessel–Paxton smearing^64^ with a width parameter of 0.1 eV. These input parameters to the plane wave DFT calculations have been established on the basis of convergence tests described in Section S2 of the Supporting Information. The calculations for the PES have been performed with a user-modified version of the Vienna ab initio simulation package^62,65^ (Vasp5.4.4) that allows calculations with a weighted average of the exchange parts of the PBE and RPBE DFs.

### 2.3 Interpolation of the PES

The H_2_-surface PES was interpolated using the corrugation reducing procedure (CRP),^49^ with the formula
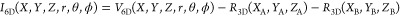
2in which *V*_6D_ is the full 6D PES of the H_2_/surface system, *I*_6D_ is the so-called 6D interpolation function of the H_2_/surface system, and *R*_3D_ is the 3D PES of the H/surface system, and (*X*_D_, *Y*_D_, *Z*_D_) are the Cartesian coordinates of H-atom D = A or B. eq 2 recognizes that most of the corrugation and the anisotropy of the H_2_-surface interaction is due to the H-atom that is closest to the surface, so that subtracting the H-atom–surface interactions from the full H_2_–surface interaction *V*_6D_ leads to the much smoother interpolation function *I*_6D_.^49^ The three-dimensional (3D) atom–surface PES is in turn written as
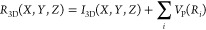
3

Equation 3 recognizes that a smoother function (the 3D interpolation function *I*_3D_) can be obtained by subtracting from the corrugated H-surface interaction the sum of pair interactions *V*_P_(*R_i_*), where *R_i_* is the distance of the H-atom to the nearest surface atoms labeled by *i*.

The interpolation procedure used for the PES of H_2_ + Al(110) is the same as used for H_2_ on Cu(110) in ref (66) where the procedure has been described in detail, albeit with respect to a coordinate system that was rotated relative to that in Figure 1A by 90°. For the interpolation of *I*_6D_, 22 configurations of (*X*,*Y*,θ, ϕ) are used, spread over five different sites (*X*, *Y*), i.e., the top site, the hollow site, the long-bridge site, the short-bridge site, and a site located halfway between the top and the hollow sites which is called the C-site (See Figure 1A), which are identical to the configurations described in ref (66).

The interpolation is done in several steps: First, for every configuration, the interpolation is performed over the *r* and *Z* degree of freedom. For this interpolation, a 22 × 17 (*r* × *Z*) grid is used, employing a two-dimensional (2D) cubic spline interpolation, over the range in *r* defined by *r*_min_ = 0.4 Å and *r*_max_ = 2.55 Å and the range in *Z* defined by *Z*_min_ = 0.0 Å and *Z*_max_ = 4.0 Å. Then, for every site, the interpolation is performed over the θ and φ degrees of freedom using symmetry-adapted sine and cosine functions. Finally, an interpolation over *X* and *Y* is performed, for which again symmetry-adapted sine and cosine functions are used. In a long range, we apply a switching function between 3.5 and 4.0 Å from the full 6D potential to a 2D asymptotic gas-surface potential that only depends on *r* and *Z*, because far away from the surface, the corrugation and anisotropy of the PES are vanishingly small. This asymptotic potential is represented by
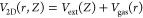
4where *V*_ext_ is a function closely describing the dependence of the PES on *Z* beyond *Z* = 3.5 Å for the BG6 geometry(see Figure 1C), and *V*_gas_ defines the H–H interaction calculated with H_2_ positioned in the middle of the vacuum. Between *Z* = 3.5 and 4.0 Å, *V*_ext_(*Z*) is positioned more or less halfway between the extremes of the full 6D interaction potential computed with the 22 different configurations (combinations of impact site and orientation), these extremes being apart by no more than 26 meV for *Z* = 3.5 Å, and by no more than 8 meV for *Z* = 4 Å. For the interpolation of *I*_3D_, the same nine sites in (*X,Y*) are used for the H-surface interaction as used in ref (66). The function *V*_P_(*R_i_*) describes the interaction of an H-atom with the surface above the top site, as used previously for the investigation of H_2_ + Cu(110).^66^

### 2.4 Calculations of Observables

The sticking probability measured in a molecular beam experiment can be computed using^14,37,46^
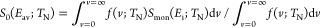
5In eq 5, *E*_av_ is the average collision energy, and *S*_mon_(*E*_i_; *T*_N_) is an intermediate quantity, which may be called the monochromatic sticking probability. To compute the sticking probability, this quantity needs to be averaged over the velocity distribution, which can be written as^67,68^
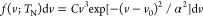
6Here, *v* is the molecule’s velocity toward the surface that is related to the incidence energy by *E*_i_ = 1/2*mv*^2^, *m* being the mass of the molecule, and the parameters characterizing the velocity distribution of the beam are the stream velocity *v*_0_ and the width parameter α, while *C* is a normalization parameter. The beam parameters used are given in Table 1. These parameters were taken from ref (46) (i.e., they were taken from Tables S5 and S6 of that paper) in which they were obtained by performing fits of TOF spectra and from a plot of the speed ratio vs the average incidence energy. The TOF spectra and the plot referred to were taken from the PhD thesis of Berger^45^ that describes experiments on H_2_ colliding with Cu(111) as well as the experiments on H_2_.
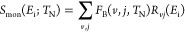
7Here, *j* is the rotational quantum number. The Boltzmann weight is given by
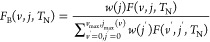
8in which
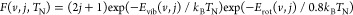
9

**Table 1:** Parameters Used for the Molecular Beam Simulations of H_2_ on Al(110)^a^

*T*_N_ (K)	*E*_av_ (kcal/mol)	*v*_0_ (m/s)	α (m/s)
1100	5.10	3679	1525
1400	7.89	3578	2550
1700	9.36	3265	3103
1120	6.00	3500	1996
1330	7.15	3555	2342
1580	8.49	3219	2903

a Al(110) we will compare with in future (A.D. Powell et al., to be published). The monochromatic sticking probability can be computed using

In eq 7, *R_vj_*(*E*_i_) is the degeneracy averaged reaction probability, i.e., the average over the (2*j* + 1) fully initial state resolved reaction probabilities (*E*_i_), where *m_j_* is the magnetic rotational quantum number (the projection of *j* on the surface normal). In eq 9, *E*_vib_ and *E*_rot_ are the vibrational and rotational energy, respectively, of the (*v*,*j*) state, and *k*_B_ is the Boltzmann constant. In these equations, it is assumed that the rotational temperature of the molecules is 0.8 times the nozzle temperature (*T*_rot_ = 0.8*T*_N_)^21,69,70^ and that the vibrational temperature is equal to the nozzle temperature *T*_vib_ = *T*_N_.^21,69^ We assume that the fractions of ortho and para-H_2_ are equal to those in the high-temperature limit and given by *w*(*j*). Then for H_2_, *w*(*j*) is equal to 1/4 for even *j* and 3/4 for odd *j*.

It is rather trivial to rewrite eqs 7–9 in terms of the fully initial state-resolved reaction probabilities. We will nevertheless provide the equations as it makes it easier to explain the procedure we use for averaging over rovibrational states in the QD calculations below. The equations are
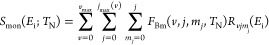
10
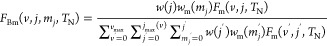
11
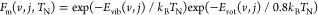
12

In having the sum over *m_j_* run from 0 to +*j* in eqs 10 and 11, we have used that *R*_*vj* – *m_j_*_(*E*_i_) = (*E*_i_), which we take into account through the weight factor *w*_m_(*m_j_*) = (2 – δ_*m_j_*,0_) in eq 11.

The integration in eq 5 and the summation in eq 7 or eq 10 can be performed in different ways. In QCT calculations, the computation of reaction or sticking probabilities always involves the selection of initial conditions using a Monte Carlo integration (or Monte Carlo averaging) procedure. If this procedure is to be used in the computation of initial sticking probabilities to select, e.g., the impact site and the initial orientation of the molecule, one might as well use Monte Carlo integration throughout in the procedure to compute *S*_0_. In this often used procedure, which may be referred to as ″full Monte-Carlo averaging″ (FMC), *S*_0_ is computed in a single calculation with the use of a Monte Carlo averaging procedure in which the initial velocity of the molecule and the initial rovibrational state are selected according to the initial conditions. This is done on the basis of an appropriate statistical procedure involving random number generation, effectively using eqs 5 and 10. If, on the other hand, the TDWP method is used to compute *S*_0_ it makes much more sense to compute the integral in eq 5 by performing a Riemann sum, because the TDWP method yields reaction probabilities over a range of closely spaced energies instead of one energy at a time.^30^ In this case, the normal procedure is to obtain results for a range of vibrational states running from *v* = 0 to *v*_max_, and from *j* = 0 to *j*_max_, where *j*_max_ may depend on *v*, and one can use either eqs 5 and 7 or eqs 5 and 10. Because in this procedure Monte Carlo averaging is used in neither the integration over incident velocity nor the averaging over initial states, we here call this procedure ″no Monte Carlo averaging″ (NMC).

A disadvantage of using the default procedure last mentioned (NMC) for QD calculations on the highly activated H_2_ + Al(110) system is that computationally expensive calculations are required for a very large number of rovibrational states. Fortunately, as we will show, it is also possible to use a partial Monte Carlo averaging procedure (PMC), in which the sum in eq 10 is performed using Monte Carlo averaging over initial rovibrational states. Specifically, rewriting eqs 10 and 11 we can then perform the sum over a much smaller number of *N*_sel_ states:
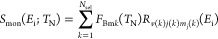
13
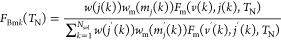
14

Equations 13 and 14 state that, in the PMC procedure we used, each rovibrational state (*v*, *j*, *m_j_* ≥ 0) included in the sum is *selected* with equal weight (i.e., without taking into account the weight factors in eqs 12 and 14) for performing a QCT or QD calculation of (*E*_i_)). Here, each state can only be selected once. The weights in eqs 12 and 14 are of course taken into account in computing *S*_0_ through eq 13, but the *N*_sel_ selected (*v*, *j*, *m_j_* ≥ 0) states all had an equal chance to be selected for use as an initial state in a dynamics calculation.

An important point is that in principle *N*_sel_, and the actual rovibrational states selected, should be the same in all beam simulations to take advantage of the feature of TDWP calculations that they provide results for a range of incidence energies, but for only one initial rovibrational state.^30^ Varying *N*_sel_ or keeping it the same but using different initial rovibrational states would lead one to either discard quantum dynamics results that are available anyhow or to perform a needlessly high number of computationally expensive QD calculations. To keep *N*_sel_ as low as possible in view of the computational cost of QD calculations, the following procedure was used. For a given number of *N*_sel_, the states to be used are generated, and the PMC value of *S*_0_, i.e., *S*_0_(PMC), is computed with QCT. If within a reasonable number of trials, we find that |*S*_0_(PMC) – *S*_0_(NMC)|/*S*_0_(NMC) < 0.1 for the beam condition corresponding to the lowest average value of *E*_i_, then the value of *N*_sel_ and the corresponding batch of states are accepted as yielding representative values for *S*_0_. Here, an assumption has been that while statistical fluctuations might lead to somewhat larger relative errors in the PMC sticking probabilities than 0.1 at somewhat higher average energies, these larger relative errors should still be of a moderate size, e.g., they should not exceed 0.2 (20%). We say this even though we assume that the reaction should be determined by the lowest amount of rovibrational states at the lowest energy beam condition, making it critical to use a high enough value of *N*_sel_ to ensure that at least some of these states are sampled. It should then be possible to obtain fairly accurate values of *S*_0_ at all relevant average incidence energies with the TDWP method on the basis of the same states in the PMC procedure with a much smaller computational effort. Below we will show that *N*_sel_ = 35 is already small enough for this purpose, while calculations on 319 (*v*, *j*, *m_j_* ≥ 0) states would have been necessary with the *v*_max_ and *j*_max_(*v*) parameters used in the NMC procedure (these parameters are collected in Table 2). An assumption used in this work is that with the rovibrational states thus selected we can also obtain QD results that are representative of NMC QD results, i.e., of the QD results that would be obtained performing QD calculations for all 319 states explicitly.

**Table 2:** *j*_max_(*v*) Parameters Determining for Which Rovibrational States  Were Taken into Account in the NMC and FMC QCT Calculations

*j*_max_(*v*), *v*=	NMC	FMC
0	15	20
1	13	20
2	11	20
3		20

In both the NMC and PMC calculations using the QCT method, we perform the Riemann sums to evaluate eq 5 for *E*_i_ in the range of 0.05–3.05 eV. Cubic spline interpolation is carried out to obtain the initial state-selected reaction probabilities for intermediate energies, and extrapolation is carried out to obtain the  for *E*_i_ < 0.05 eV. Tests showed that the upper bound in eq 5 can be replaced by a value of the velocity corresponding to *E*_i_ = 2.20 eV, although the actual upper bound corresponded to 3.05 eV. QD calculations were only carried out up to *E*_i_ = 1.05 eV; to obtain QD results, for higher values of *E*_i,_ we simply used the QCT reaction probabilities computed for these energies.

### 2.5 Dynamics Methods

#### 2.5.1 Quantum Dynamics

The time-dependent wave packet (TDWP) method^30^ as implemented in our in-house developed code^31,36^ was used to solve the time-dependent Schrödinger equation (TDSE)
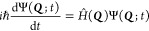
15

In eq 15, the six molecular coordinates described in Section 2.1 are given by the vector ***Q***. Ψ(***Q***; *t*) is the time-dependent nuclear wave function of the system and *Ĥ*(***Q***) is the time-independent Hamiltonian given by
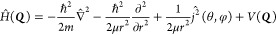
16Here, μ is the reduced mass of H_2_, ∇̂ and *ĵ* are the nabla operator acting on the center-of-mass coordinates of the molecule and the angular momentum operator, respectively, and *V*(***Q***) is the 6D interpolated CRP PES.

To solve the TDSE, an initial wave function is set up as a product of a Gaussian wave packet *u*(*Z*_0_, *k*_0_^*Z*^) centered on *Z*_0_ with average momentum *k*_0_^*Z*^, a two-dimensional plane wave function ϕ(*k*_0_^*X*^, *k*_0_^*Y*^) for motion along *X* and *Y*, a vibrational wave function ψ_ν, *j*_(*r*), and a rotational wave function *Y*_*j*, *m_j_*_(θ, ϕ) of incident H_2_:
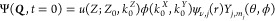
17

In eq 17, the two-dimensional plane wave function and the Gaussian wave packet are defined as
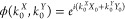
18
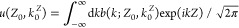
19with
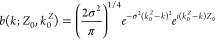
20Here, σ is the width of the wave packet for motion in *Z* centered around the initial average momentum *k*_0_^*Z*^, and *k*_0_^*X*^ and *k*_0_^*Y*^ are the initial momenta for motion along *X* and *Y*, which are taken equal to zero here to describe normal incidence. As described in more detail in the Supporting Information, the width σ is chosen in such a way that 90% of the Gaussian wave packet is placed in an energy range *E*_i_ ∈ [*E*_min_, *E*_max_], and four of these energy ranges can be used to generate results between *E*_i_ = 0.05 and 1.05 eV. In the expression for the time-dependent wave function, a Fourier representation was used to represent the dependence of the wave function on *Z*, *r*, *X*, and *Y*, and fast Fourier transforms were used to evaluate the action of the corresponding kinetic energy operators on the wave function.^71^ We employed a finite basis representation to represent the angular part of the wave function.^72,73^eq 15 is solved numerically using the split operator method^74^ using a time step Δ*t*. A complex absorbing potential (CAP, actually, a negative imaginary potential of quadratic order^75^) is used to absorb the reacted and scattered wave packet for large values of *r* and *Z*, respectively. For high incidence energies relative to the reaction threshold, the scattered fraction of the wave function is analyzed through the scattering amplitude formalism,^76,77^ after which state-to-state scattering probabilities *P*_sc_ can be obtained from the squares of the corresponding S-matrix elements.^31,36^ Summing the *P*_sc_ and subtracting from 1 then yield the fully initial state-resolved reaction probability (*E*_i_).

For incidence energies just above and below the reaction threshold for the initial (*v*,*j*) state, we have used the flux-analysis method^78,79^ to compute (*E*_i_) more directly, by analyzing the reactive flux through the five-dimensional surface at a large enough and fixed value of *r* = *r*_fl_ = 6.55 *a*_0_ ≈ 3.47 Å. This H–H distance is far beyond that of the barriers to DC for the system investigated (see Section 3.1 below) but lower than the value where the CAP absorbing the reacted part of the wave packet is turned on (see Table S4 of the Supporting Information). Also, we have checked that using this value yields results equal to those of the scattering amplitude formalism in the regime where results of the latter method are not affected by resonances. We found that the calculation of the state-to-state scattering probabilities (and, thereby, of the (*E*_i_)) at *E*_i_ near the reaction threshold may be hampered by the formation of meta-stable states located in the entrance channel when using the scattering matrix formalism. This was not the case with the flux-analysis formalism, presumably because these entrance channel states do not affect the reaction due to the high barrier (of about 1 eV) to the reaction; instead, they only affect the scattering back to the gas phase.

The input parameters to the TDWP calculations were selected on the basis of convergence tests. These parameters are discussed in Section S4 and provided in Table S4 of the Supporting Information.

#### 2.5.2 Quasi-Classical Dynamics

In performing the classical dynamics calculations, we always impart the zero-point energy to the vibration of H_2_, i.e., we use the QCT method.^28,29^ To evaluate the initial state-resolved reaction probabilities, we placed our molecule initially at *Z* = 8.0 Å with a velocity normal toward the surface that corresponds to a specific initial incidence energy. At this distance, the interaction of the molecule with the surface is essentially zero. For each initial rovibrational state modeled, calculations were performed for fixed incidence energies in the range of 0.05–3.05 eV. For each energy and initial rovibrational state, typically *N*_T_ = 500,000 trajectories were computed. In all cases, the maximum propagation time is 2 ps. In the calculations using the FMC procedure, *N*_T_ = 190,000,000 trajectories were run for each initial condition. In the FMC procedure states with *v* up to 3 were included, and the *j*_max_ values employed with the vibrational states are listed in Table 2.

To propagate the equation of motions, the Bulirsch–Stoer method was used.^80,81^ As in the TDWP calculations, the time-independent Schrödinger equation (TISE) was solved using the Fourier grid Hamiltonian method^82^ to determine the bound state rotational-vibrational eigenvalues of gas-phase H_2_. The bond distance and the vibrational velocity of the molecule are randomly sampled from a one-dimensional quasi-classical dynamics calculation of a vibrating H_2_ molecule for the corresponding rovibrational energy.^44^ The orientation of the molecule, given by θ and φ, is chosen based on the selection of the initial rotational state. The magnitude of the classical initial angular momentum is fixed by  and its orientation, while being constrained by , is otherwise randomly chosen as described by Wijzenbroek et al.^83^ Here, Θ_l_ is the angle between the angular momentum vector ***j*** and the surface normal. Other initial conditions are randomly chosen as described in ref (44). Reaction is defined to occur if the H–H distance becomes longer than 2.2 Å. The initial state-selected reaction probability can be computed as
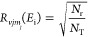
21where *N*_r_ is the number of reacted trajectories. The statistical error in the computed reaction probability, which defines a 68% confidence interval, can be computed as
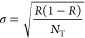
22

The reaction probabilities can be used to compute *S*_0_ using eqs 5 and 7 or 5 and 13 as described above in Section 2.4.

## 3 Results and Discussion

### 3.1 Fitted Potential Energy Surface

**Figure 2:**
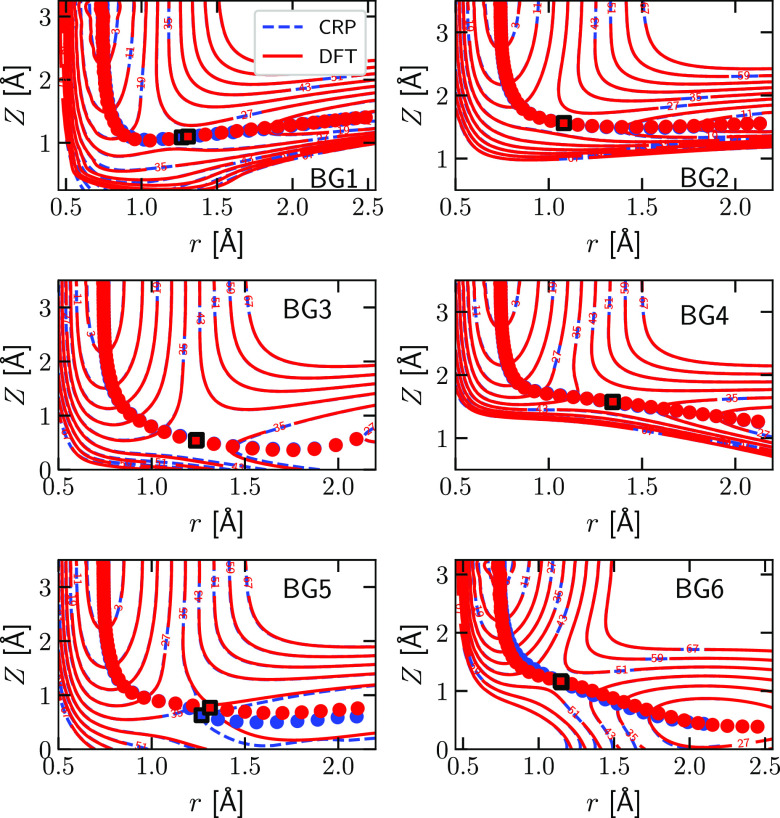
Elbow plots of the H_2_-Al(110) PES as directly calculated with DFT (red solid lines) and fitted with the CRP (blue dashed lines). The blue numbers label the contour lines of the PES. Red (blue) circles indicate the minimum energy path from reactants to product as computed directly with DFT (obtained from the CRP fit). The red (blue) square indicates the position of the barrier in 2D as computed with DFT (interpolated with the CRP). Results are given for the six barrier geometries indicated in Figure 1C and investigated in ref (41).

The accuracy of our CRP 6D potential has been checked by comparing the interpolated results to the raw DFT data. Figure 2 shows, for six selected high symmetry configurations (BG1-BG6, called TS1-TS6 in Table 5 of ref (41) see also Figure 1C), 2D cuts through the PES (also called elbow plots). In all cases, H_2_ was oriented parallel to the surface. The CRP reproduces the DFT data quite well. Moreover, the 2D minimum energy paths (MEPs) obtained with the CRP are in close agreement with the DFT results (Figure 2). Furthermore, a quantitative comparison between the CRP and the DFT results is shown in Table 3 for all the BGs represented in Figure 2. As can be seen, the barrier heights and geometries derived from the CRP are in excellent agreement with the raw DFT results. The mean absolute deviation (MAD) associated with the six barrier heights is just 0.24 kcal/mol.

**Table 3:** Comparison between CRP and DFT of the Barrier Heights (in kcal/mol) and Locations (*r*_b_, *Z*_b_) (in Å), Relative to the Gas-Phase Minimum, for All the Six BGs (See Figure 1C and Table 5 of ref (35))^a^

	BG1	BG2	BG3	BG4	BG5	BG6
*Z*_b_^DFT^	1.08	1.56	0.54	1.57	0.77	1.15
*Z*_b_^CRP^	1.08	1.56	0.55	1.57	0.63	1.15
*r*_b_^DFT^	1.22	1.08	1.24	1.34	1.31	1.15
*r*_b_^CRP^	1.26	1.08	1.24	1.34	1.27	1.15
*E*_b_^DFT^	25.3	24.8	37.5	37.8	34.8	49.4
*E*_b_^CRP^	25.40	24.78	37.73	38.03	35.60	49.44
Δ*E*	0.10	–0.02	0.23	0.24	0.80	0.05

a All geometries are for the H_2_ molecule lying parallel to the surface (θ = 90°). Also indicated are the signed errors between DFT and CRP (Δ*E* = *E*_b_^CRP^ – *E*_b_^DFT^).

### 3.2 Sticking Probabilities Computed with Quantum and Quasi-Classical Methods, and Their Comparison

As a “sanity check”, we first performed a comparison of the *S*_0_ computed with the NMC procedure and the FMC procedure. The results show that the *v*_max_ and the (*j*_max_(*v*),*v* = 0–2)) values used in the NMC procedure (see Table 2) were high enough to yield, for the range of molecular beam conditions investigated here, values of *S*_0_ that are converged with respect to the number of rovibrational states included, and accurate enough for our purposes (see Figure S1 of the Supporting Information).

**Figure 3:**
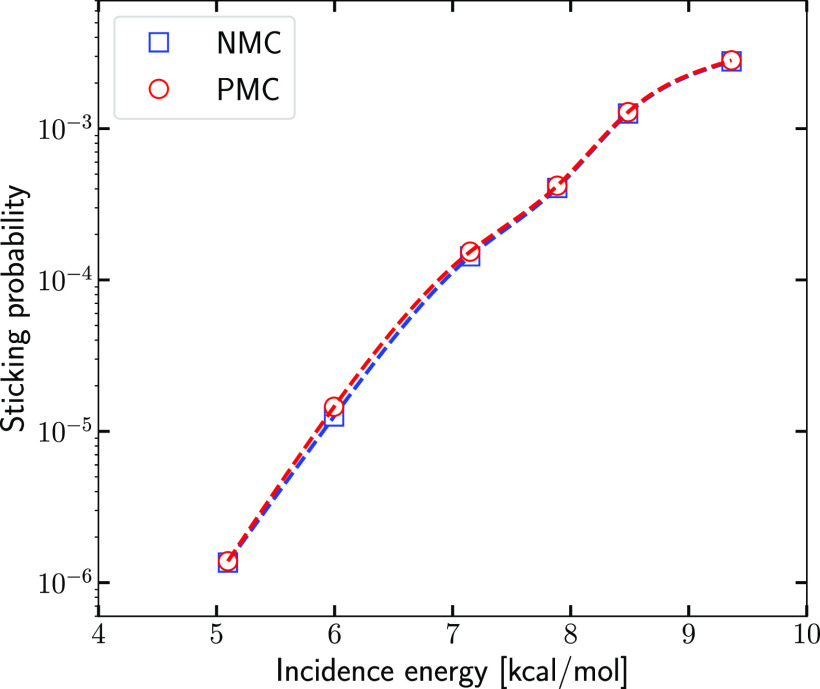
Sticking probabilities computed with the QCT method using averaging over all 319 *v* = 0, 1, and 2 (*v*, *j*, *m_j_*) states (“NMC”) and Monte Carlo averaging over only 35 such states (“PMC”).

We next investigated the accuracy of the PMC procedure by comparing QCT results obtained with the NMC and the PMC procedures (see Figure 3). Even though the decisions on which the number of states to be included, and which states to be included in the PMC procedure were taken only on the basis of the results for the lowest *E*_i_, we find that the PMC results are accurate enough for our purpose for all average *E*_i_. The absolute value of the relative error was 1.6% for ⟨*E*_i_⟩ = 5.1 kcal/mol (meeting our requirement that it should be lower than 10%), 15.9% for ⟨*E*_i_⟩ = 6.0 kcal/mol, and decreased monotonically from 7.4% for ⟨*E*_i_⟩ = 7.1 kcal/mol to 1.6% for ⟨*E*_i_⟩ = 9.4 kcal/mol (meeting our requirement that it should in no case be higher than 20%). The nonmonotonic dependence of the relative error on average incidence energy can be attributed to statistics: the selected batch of states is good for ⟨*E*_i_⟩ ≥ 7.1 kcal/mol, good enough for the lower value of 6.0 kcal/mol, and perhaps by chance it is excellent for the lowest value (5.1 kcal/mol), where the reactivity should be dominated by the smallest amount of rovibrational states that are thermally populated. We conclude that the value used for *N*_sel_ (i.e.,*N*_sel_ = 35) is high enough for our purposes, that the batch of rovibrational states selected is good enough to yield representative QCT results, and that it should therefore in principle suffice to base the QD-QCT comparison on the results for this batch of rovibrational states only.

**Figure 4:**
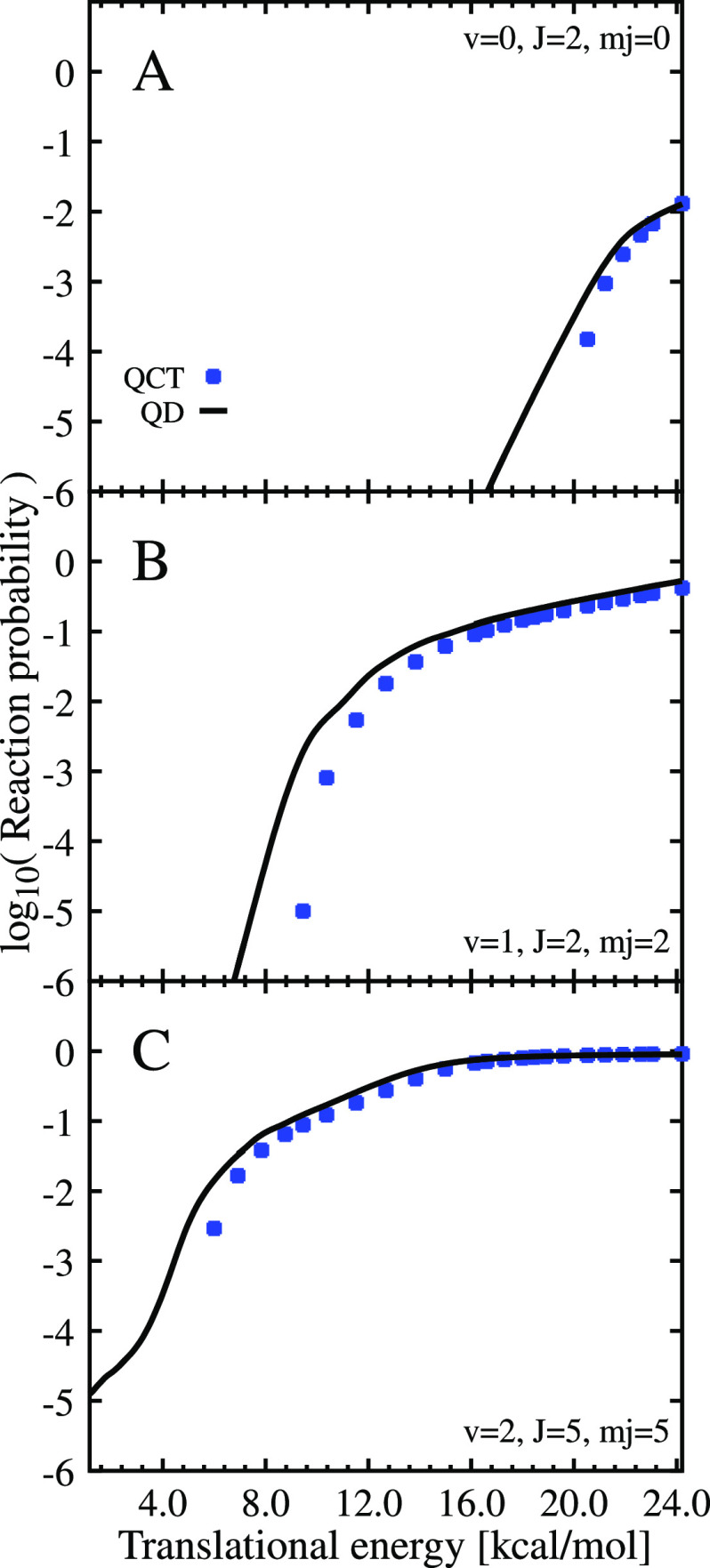
(*E*_i_) computed with QD (black line) and QCT (blue squares) dynamics are compared for three different initial rovibrational states, of which (A) one with *v* = 0, (B) one with *v* = 1, and (C) one with *v* = 2.

We compare (*E*_i_) computed with QD and QCT dynamics for three initial rovibrational states (with *v* = 0, 1, and 2, respectively, with these three states contributing to the *S*_0_ computed with the PMC procedure) in Figure 4A–C. The trends we see in typical comparisons of QD and QCT results for specific initial rovibrational states are exemplified in this plot. We usually see that the differences between the (*E*_i_) computed with QD and QCT dynamics become increasingly small for *E*_i_ increasing and approaching the reaction threshold for the specific initial rovibrational state. This reaction threshold is close to ≈23.8 kcal/mol = 1.03 eV for the (*v* = 0, *j* = 2) states for which results are presented in Figure 4A, the minimum barrier height of the CRP potential being 24.8 kcal/mol), while it is lower for the other initial states included in the sum of eq 10, which have higher rovibrational energies. At lower energies we typically see QD reaction probabilities that are higher than the QCT results, which we attribute to tunneling. The opposite is also sometimes observed (see the result in Figure 4A for the highest *E*_i_), which is most likely due to artificial zero-point energy conversion (in the QCT method conservation of zero-point energy during the trajectory is not guaranteed^29^).

**Figure 5:**
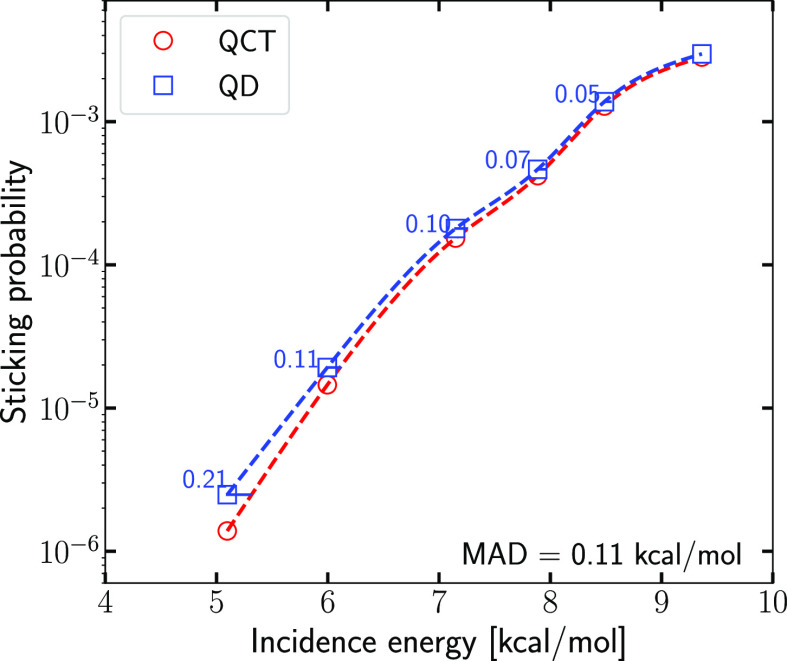
Sticking probabilities computed with QD (blue squares) and with QCT (red circles) dynamics using the PMC procedure are compared. The distances along the energy axis (in kcal/mol) between the QD sticking probabilities and the spline interpolated QCT sticking curve are also presented in blue.

Figure 5 shows the effect of the differences between QD and QCT values of (*E*_i_) on the *S*_0_ curves computed with QD and with QCT dynamics with the PMC procedure. Especially at low ⟨*E*_i_⟩_,_ the QD *S*_0_ are considerably larger than their QCT counterparts (by 80 and 32% at ⟨*E*_i_⟩ = 5.1 and 6 kcal/mol, respectively, decreasing monotonically to only 5% at 9.4 kcal/mol). As a result, the QD *S*_0_ curve is shifted toward lower values of the average incidence energy than the QCT curve, with the value of the energy shift decreasing toward larger ⟨*E*_i_⟩. As a result of the decreasing value, the difference in the curves cannot really be quantified well with the single average value of the energy shift (0.11 kcal/mol). The computed energy shifts are substantially smaller than the accepted criterion of chemical accuracy (which is 1.0 kcal/mol). Nevertheless, it should still be good to correct for quantum effects and artifacts of the QCT method (i.e., problems with zero-point energy conversion, and, more generally, lack of quantization once a trajectory has been started) when comparing with experiment, to enable maximally reliable conclusions regarding the accuracy of the electronic structure approach used. However, it is important to note that quantum corrections to the sticking curve are not likely to have a big effect on the evaluation of the accuracy of an electronic structure method through the usual procedure, in which computed and measured sticking probability curves are obtained and the accuracy of the barrier is judged on the basis of the energy shift between these curves.^14,46,84,85^

### 3.3 Analysis of the Size of the Quantum Effects on the Sticking: Role of Vibration and Incidence Energy

**Figure 6:**
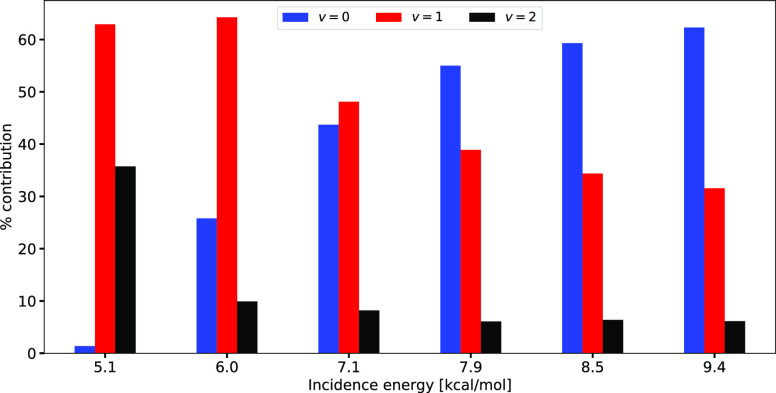
Histogram of the percentage contributions to *S*_0_ of H_2_ in its *v* = 0 (blue bars on the left), *v* = 1 (green bars in the middle), and *v* = 2 (red bars on the right) vibrational states to the sticking at the six different average incidence energies shown.

It is interesting to note that the energy shifts between the QD and QCT curves in Figure 5 appear as rather small. From this point of view, the quantum effects on the sticking probability appear to be rather small at *E*_i_ much smaller than the minimum barrier to DC (24.8 kcal/mol). To explain this, we have analyzed our NMC QCT results in some detail. We have looked at the effect of the distributions of the initial vibrational state of H_2_ and of its *E*_i_, where both are ultimately governed by the *T*_N_ used in the experiments using pure beams, as employed for H_2_ + Al(110).^20^Figure 6 shows the percentage contribution to *S*_0_ of H_2_ in specific initial vibrational states for the average incidence energies investigated here. At the lowest ⟨*E*_i_⟩ (5.1 eV), the sticking is dominated by *v* = 1 H_2_, and the sticking of *v* = 2 H_2_ is much more important than the sticking of *v* = 0 H_2_. The reaction of *v* = 1 H_2_ also dominates the sticking at ⟨*E*_i_⟩ = 6.0 and 7.15 eV, and it remains important even at higher *E*_i_. Part of the reason for the small quantum effects as perceived here may therefore be that a small fraction of the H_2_ molecules is incident in high initial vibrational states, which may help them to react in a classical “over the barrier” fashion without the need for tunneling. We note that experiments were able to show that the activated sticking of H_2_ on Cu(111) is dominated by DC of H_2_ in its *v* = 1 state, and at very low *E*_i_ by DC of H_2_ in its *v* = 2 state,^21^ and that this might likewise explain why differences between QCT and QC values of *S*_0_ were found to be small for this system as well.^32^ Similarly, calculations on experiments on D_2_ + Ag(111) (with an even higher barrier of 31.8 kcal/mol) suggested that the sticking in this system should be dominated by *v* = 3 D_2_ for ⟨*E*_i_⟩ = 11.2 kcal/mol.^86^ Note that the extent to which the sticking is dominated by the contribution of the molecule in a particular vibrational state should also depend on the distribution of the translational energy of the incident beam, and not just on the characteristics of the DC system itself.

**Figure 7:**
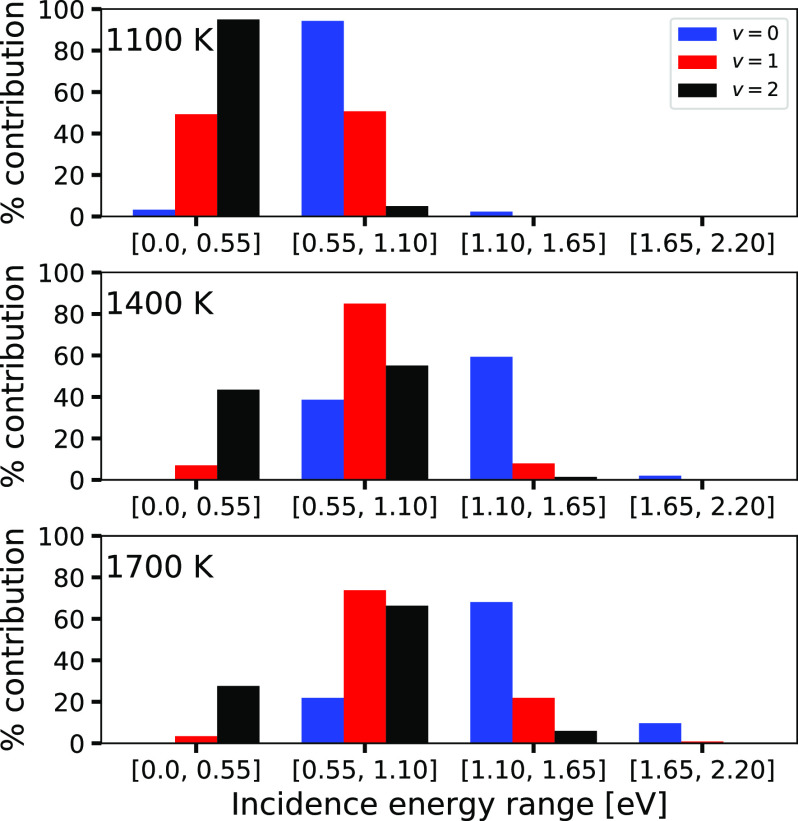
Histogram of the percentage contributions of H_2_ incident in four ranges of incidence energies to the reaction of H_2_ in a particular initial vibrational state *v*, for *v* = 0 (red bars on the left), *v* = 1 (green bars in the middle), and *v* = 2 (blue bars on the right) in the sticking at three different nozzle temperatures.

**Figure 8:**
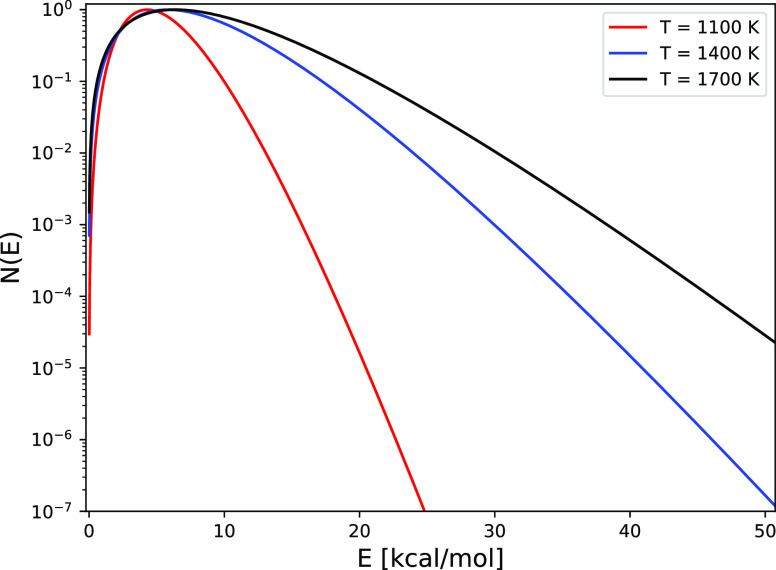
Flux-weighted translational energy distributions corresponding to the velocity distribution expression of eq 6, as determined from ref (45) for the conditions corresponding to *T*_N_ = 1100, 1400, and 1700 K (⟨*E*_i_⟩ =5.1, 7.9, and 9.4 kcal/mol). The distributions have been rescaled to make their maximum coincide with 1.0.

The role of *E*_i_ is addressed in more detail in Figure 7. For three values of *T*_N_ (corresponding to ⟨*E*_i_⟩ = 5.1, 7.9, and 9.4 kcal/mol), the percentage contribution to the sticking of H_2_ in a particular vibrational state *v* is shown of the H_2_ molecules incident in four ranges of *E*_i_, which are indicated in eV. The minimum barrier height in the PES (24.8 kcal/mol) is approximately equal to 1.08 eV. At *T*_N_ = 1400 and 1700 K, most *v* = 0 molecules react with *E*_i_ in excess of the TS energy (i.e., at *E*_i_ > 1.10 eV), and most *v* = 1 molecules react with *E*_i_ > 0.55 eV. At these conditions, most of the *v* = 0 molecules that react in the QCT calculations are thus able to do so in a classical fashion with an incidence energy that exceeds the classical barrier height. That they are able to do so is related to the widths of the translational energy distributions in the experiments. As shown in Figure 8, the 1400 and 1700 K beams still have a considerable fraction (relative to the computed values of *S*_0_, see Figure 3) of molecules in them with *E*_i_ exceeding the classical barrier height of 24.8 kcal/mol.

Whereas at *T*_N_ = 1400 and 1700 K most *v* = 0 molecules react with *E*_i_ > 1.1 eV, most *v* = 1 and *v* = 2 molecules react at smaller *E*_i_ under these conditions (see Figure 7). The extent to which the sticking is dominated by the DC of H_2_ in a particular vibrational state, and how this is related to incidence energy, depends on how efficiently pre-exciting the vibration of H_2_ promotes reaction relative to enhancing *E*_i_. This can be expressed by the vibrational efficacy, which can be defined through^22,37^
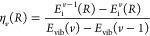
23

**Figure 9:**
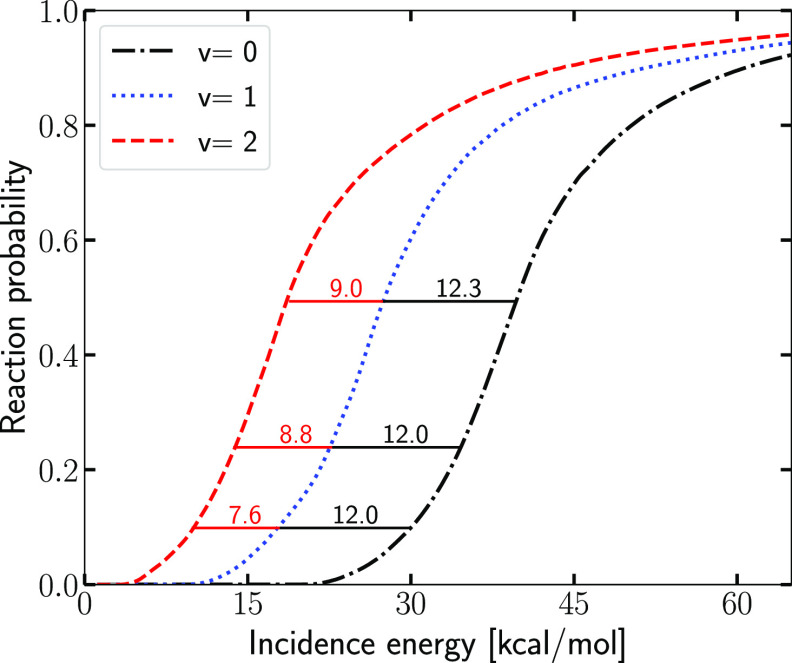
Initial state-selected reaction probability for the (*v*,*j* = 0) state of H_2_ is shown as a function of *E*_i_ for *v* = 0, 1, and 2, indicating energy spacings between the curves (in kcal/mol) for values of the reaction probability of 0.1, 0.25, and 0.5.

In eq 23, *E*_i_^*v*^(*R*) is the incidence energy for which *R*_*v*, *j* = 0_(*E*_i_) first becomes equal to *R*, and *E*_vib_(*v*) is the vibrational energy of the molecule in the state *v*. To illustrate the vibrational efficacy in the H_2_ + Al(110) system, we show *R*_*v*, *j* = 0_(*E*_i_) in Figure 9 for *v* = 0, 1, and 2, and vibrational efficacies are presented in Table 4. For *v* = 1 the vibrational efficacy exceeds 1, although it is smaller than the vibrational efficacy computed for D_2_ on Ag(111) for *R* = 0.24, which was equal to 1.37.^86^ As also discussed for D_2_ + Ag(111) in ref (86) and analyzed in detail in ref (87), vibrational efficacies >1 show that, in the lower vibrational state (here in *v* = 0), the molecule has to come in at such high *E*_i_ in order to react that it cannot follow the minimum energy path and skids off it.^88,89^ As a result, it must cross the “mountain pass” from the reactant to the product “valley” at a higher point than the optimum, lowest one. This effect has previously been called the bobsled effect.^90^ The high efficiency with which *v* = 1 H_2_ is able to react on Al(110) helps with explaining why quantum effects on the sticking in this system are so small for low *S*_0_: thanks to the high vibrational efficacy *v* = 1 H_2_ can react in a classical over the barrier type fashion at relatively low incidence energies. An additional reason that quantum effects on the sticking should be relatively small for the experiments performed by Rendulic and co-workers is that the translational energy distributions of their molecular beams are broad,^45^ also when compared to H_2_ beams used by other groups, as discussed in refs (46, 91). As a result, especially at higher *T*_N_, a sizable fraction of the incident molecules in the high-energy tail of the beam have a high enough *E*_i_ to react in a classical fashion.

**Table 4:** Vibrational Efficacies for DC of H_2_ on Al(110)

	η_*v*_(*R* = 0.1)	η_*v*_(*R* = 0.25)	η_*v*_(*R* = 0.5)
*v* = 1	1.01	1.01	1.03
*v* = 2	0.67	0.78	0.80

## 4 Conclusions

We evaluate the accuracy of the QCT method, or, alternatively, the importance of quantum effects for the sticking of H_2_ on Al(110), for conditions that should be close to the conditions under which molecular beam experiments have been done on this system.^20^ For this purpose, QCT and QD calculations have been done with the BOSS model on a PES obtained with DFT, which exhibits a minimum barrier height close to that recently obtained with QMC calculations.^41^ To keep the number of QD calculations to be performed small, a procedure (PMC) was used in which Monte Carlo averaging over the initial rovibrational states of H_2_ was employed. This procedure allowed the quasi-classical calculation of sticking probabilities with a relative error <7.5% for 5 of the six initial conditions investigated, and of 16% for one of these conditions, at approximately an order of magnitude less computation time.

The sticking probabilities computed with QD using the PMC procedure exceed the ones computed with the QCT method by 80 and 30% for the two beam conditions corresponding to the lowest incidence energies (5.1 and 6.0 kcal/mol), decreasing to only 5% for the highest incidence energy of 9.4 kcal/mol. The sticking probability curve computed with QD is shifted to lower energies relative to the QCT curve by 0.21 to 0.05 kcal/mol, with the highest shift obtained for the lowest incidence energy. The quantum effect in the form of this energy shift may be viewed as being rather small for molecular beam sticking experiments in which the average incidence energies (5.1–8.5 kcal/mol) are much smaller than the minimum barrier height of the system investigated (24.8 kcal/mol). The smallness of the quantum effects is explained on the basis of the large vibrational efficacy of the system (>1 for *v* = 1) and of the broadness of the translational energy distributions of the molecular beams used in the experiments we address, which mean that sticking can take place through DC of vibrationally excited molecules in the high incidence energy tails of the molecular beams. We conclude that ″quantum effects″ are not expected to play a major role in calculations that would evaluate the accuracy of electronic structure methods for determining the minimum barrier height to DC for H_2_ + Al(110) on the basis of existing molecular beam experiments, as the verdict would depend on the energy shift between the computed and the measured sticking probability curve. However, for maximum reliability of the conclusions on accuracy it would still be good to take the quantum effects into account, as the maximum shift (0.21 kcal/mol) is not negligible on the scale of ″chemical accuracy″ (1 kcal/mol).

## Supporting Information Available

Supporting text on the setting up of the metal slab modeling the surface, convergence of the molecule–surface interaction energies with DFT input parameters, and on the input to the TDWP calculations (Sections S1–S4), parameters characterizing the slab modeling the Al(110) surface (Table S1), results of convergence tests Table S2, parameters characterizing the DFT calculations for the PES (Table S3), input parameters to the TDWP calculations (Table S4), and comparison of quasi-classical sticking probabilities obtained with the FMC and NMC procedures (Figure S1) (PDF)
