# Supplementary material for: Simulating Highly Activated Sticking of H2 on Al(110): Quantum versus Quasi-Classical Dynamics
Source: J Phys Chem C Nanomater Interfaces. 2023 Mar 14;127(11):5395–407. doi: 10.1021/acs.jpcc.3c00426 (PMC10041643; doi:10.1021/acs.jpcc.3c00426)
Supplement: Supplementary file 1 — jp3c00426_si_001.pdf [file jp3c00426_si_001.pdf]

Supporting Information to: Simulating Highly Activated Sticking of H_2 on Al(110): Quantum versus Quasi-Classical Dynamics.

Theophile Tchakoua[†], *Andrew D. Powell*[†], *Nick Gerrits*[†⊥], *Mark F. Somers*[†], *Katharina Doblhoff-Dier*[†], *Heriberto F. Busnengo*[‡*], *and Geert-Jan Kroes*[†*]

[†]Leiden Institute of Chemistry, Gorlaeus Laboratories, Leiden University, P.O. Box 9502, 2300 RA Leiden, The Netherlands

[‡]Instituto de Física Rosario (IFIR), CONICET-UNR, Bv. 27 de Febrero 210 bis (2000) Rosario, Argentina, and Facultad de Ciencias Exactas, Ingeniería y Agrimensura, UNR, Av. Pellegrini 250 (2000) Rosario, Argentina

S1. Setting up a 0 K metal slab for the convergence tests of the calculations with density functional theory.

All calculations with density functional theory (DFT) used for setting up the metal slab and for performing convergence tests for computing the molecule-metal surface interaction have been performed with the VASP 5.3.5 code[1-2].

Calculations of H_2 - Al(110) interaction energies are preceded by DFT calculations to set up the slab modeling the Al(110) surface according to the specific reaction parameter density functional (SRP DF) used. First, the bulk lattice constant a_l has been determined using a bulk lattice relaxation calculation. This calculation used a 1 x 1 x 1 super cell, a plane wave cutoff energy of 300 eV, 2nd order Methfessel-Paxton smearing[3] with a smearing energy of 0.1 eV, and 25 x 25 x 25 Γ-centered k-points. Also, the calculations used projected augmented wave (PAW) pseudo-potentials (PP) that were somewhat harder than the ordinary PAW PP available from VASP (i.e., we used the PP labeled Al_GW, with date stamp 19 March 2012, of VASP) as calculations with vdW-DF correlation functionals require somewhat harder PP than calculations with semi-local density functionals (DFs) within the generalized gradient approximation (GGA) would normally need[4-7]. Next, the distances between layers n and n+1 $d_{n,n+1}$ (n=1 defines the surface layer) 10-layer Al slab (see below for the choice of 10 layers) in the slab were allowed to relax. These calculations employed the same PP as used to determine the bulk lattice constant, a 1 x 1 super cell, a plane wave cutoff energy of 300 eV, 2nd order Methfessel-Paxton smearing[3] with a smearing energy of 0.1 eV, 25 x 25 x 1 Γ-centered k-points, and a vacuum distance of 24 Å. The resulting "0 K" parameters are provided and compared with experiment in Table S1. Here, the experimental data for 0 K have been extracted from experiments presented in Ref.[8] as described in the Supporting Information of Ref.[9]. The 0 K experimental data in Table S1 have not been corrected for zero-point anharmonic expansion effects (they are the data from table S5 of Ref.[9],

where the bulk lattice constant was obtained by multiplying the bulk interlayer distance determined in Ref.[8] with $2\sqrt{2}$).

S2. DFT convergence tests.

The input parameters to the DFT calculations of the interaction energies were based on convergence tests for the BG1 and BG2 geometries of (Fig.1) (i.e., the geometries labeled TS1 and TS2 in Ref.[9]). These tests were performed using a slightly different functional as used in the calculation of the potential energy surface (PES), i.e., in Eq.1 68% RPBE[10] and 32% PBE[11] exchange was used in the convergence tests. The conclusions from the tests on BG2 were similar to those for BG1, therefore we only present results for BG1. The number of Al layers used in the Al slab to model the Al(110) surface was set to 10 in all tests, as previous convergence tests with the PBE DF had shown that DFT calculations with a 10-layer slab produced results that are very similar to the converged results obtained with a 19 layer slab[9]. For Al the calculations used the same PP as mentioned above for setting up the metal slab, and PAW PP for H that were somewhat harder than the ordinary PAW PP available from VASP (i.e., the PP labeled H_GW with date stamp 21 April 2008, of VASP), as calculations with vdW-DF correlation functionals require somewhat harder PP than calculations with semi-local GGA DFs would normally need[4-7]. The calculations used a 3D Al lattice constant that was computed self-consistently with the DF used in the tests, and the same was true for the interlayer distances employed. The tolerance parameter governing the convergence of the DFT energy with electronic iteration number (EDIFF in VASP) was set to 10^{-6} eV.

We first tested the convergence of the DFT results with the size of the surface unit cell (test series 1 in Table S2). Going from the (N_c x N_c) = (2x2) to the (3x3) surface unit cell changed the BG1 energy by no more than 0.2 kcal/mol. We chose the tighter (3x3) setting.

In a second step (series 2 in table S2), we tested convergence with respect to the plane-wave cut-off E_{PW}. Using E_{PW} = 540 eV yields results that differ from results obtained with 700 eV by no more than 0.15 kcal/mol. We therefore adopted a value of 540 eV for E_{PW}.

In the third step (series 3 in Table S2), we tested the number of k-points N_k required in the plane wave DFT calculations, using Monkhorst-Pack[12] k-point integration. (Is that correct, in the main paper it says we used a Γ- centered grid of k-points, is that the same?). Tests show that the BG1 energy changes by no more than 0.09 kcal/mol going from (N_k x N_k) = (8x8) k-points to (N_k x N_k) = (14x14) with the use of a (3x3) surface unit cell. We therefore chose to use a setup with (8x8) Monkhorst-Pack k-point integration.

We next (series 4 in Table S2) tested the convergence with respect to the vacuum distance D_v. Using D_v = 16 Å yields results converged to within about 0.05 kcal/mol, and we therefore chose to use this value for D_v.

Finally, we tested the convergence with respect to the energy smearing value E_{sm} used with first order and second order Methfessel-Paxton smearing[3] (series 5 in Table S2). With either order the BG1 energy changes by less than 0.05 kcal/mol going from E_{sm} = 0.2 to 0.1 eV. Also, the results for E_{sm} = 0.1 eV are very similar for order 2 and order 1. We therefore chose to use E_{sm} = 0.1 eV with first order Methfessel Paxton smearing.

The set up constituted by the input parameters to VASP is summarized in Table S3.

S3. Setting up a 220 K slab for the computation of the potential energy surface.

To compute the DFT data on which the PES was based, we used a set up that is appropriate for the surface temperature T_s for which the dynamics calculations were performed, which was taken

equal to the T_s used in the experiments (220 K[13]). Already in Ref.[9], we determined the experimental bulk interlayer distance at 0 K by extrapolating the measured temperature dependent bulk interlayer distance to 0 K, yielding a value of 1.4239 Å (we used the data in fig.6 of Ref.[8] for this, see also Table S1). Multiplying this value with $2\sqrt{2}$ yields a 0 K bulk lattice constant of 4.0274 Å. Next, we determined the slope of the temperature dependent bulk interlayer distance from the experimental data, yielding 4.3 x 10^{-5} Å K^{-1} (again, we used the data in fig.6 of Ref.[8] for this). Using these two data yields a value of 1.4335 Å for the experimental bulk interlayer distance at 220 K. Multiplying this value with $2\sqrt{2}$ yields an experimental 220 K bulk lattice constant of 4.0544 Å.

We then obtain the 220 K "DFT lattice constant" using

$$a_l^{DFT}(220\text{ K}) = a_l^{DFT}(0\text{ K})\frac{a_l^{\exp}(220\text{ K})}{a_l^{\exp}(0\text{ K})} \qquad \text{(S3.1).}$$

Substituting the 0 K lattice constant obtained from DFT (of 4.08708 Å, see Table S1), we obtain a value of 4.11448 Å for $a_l^{DFT}(220\text{ K})$. In an entirely analogous fashion, a 220 K DFT bulk inter layer distance $d_{n,n+1}^{DFT}(220\text{ K})$ of 1.4547 Å is obtained.

We next obtained interlayer distances between layers 1 and 2, and between layers 2 and 3, from our DFT calculations and the experiments of Ref.[8]. First we determined experimental 0 K interlayer distances using extrapolation of the temperature dependent interlayer distances displayed in fig.6 of Ref.[8], yielding $d_{1,2}^{\exp}(0\text{ K})$ = 1.3409 Å. From the slope (-3.2 x 10^{-5} Å K^{-1}) we then compute $d_{1,2}^{\exp}(220\text{ K})$ = 1.3339 Å. Using the relaxed interlayer distance from the DFT slab calculation of 1.3579 Å and an equation analogous to Eq.S3.1 we then obtain $d_{1,2}^{DFT}(220\text{ K})$ = 1.3508 Å. The analogous procedure for the interlayer distance between layers 2 and 3 yields $d_{2,3}^{DFT}(220\text{ K})$ = 1.5075 Å, using $d_{2,3}^{\exp}(0\text{ K})$ = 1.4824 Å, a slope of (8.2 x 10^{-5} Å K^{-1}), and a relaxed interlayer distance from the DFT slab calculation of 1.4894 Å. The experiments of Ref.[8] did not

provide reliable values of how, in the deeper lying layers, the interlayer distances of $d_{3,4}$, $d_{4,5}$, and $d_{5,6}$ change with temperature. We therefore assume that these can be obtained from the bulk interlayer distance DFT value $d_{n,n+1}^{DFT}(220\text{ K})$, the percentage changes of these interlayer distances in the relaxed 0 K DFT slab, and $d_{n,n+1}^{DFT}(0\text{ K})$. The percentage changes of these 0 K interlayer distances, which are tabulated in Table S1, were $\Delta(d_{3,4})$ = -3.2%, $\Delta(d_{4,5})$ = 1.9%, and $\Delta(d_{5,6})$ = 0.8, respectively. The resulting 220 K values of the corresponding interlayer distances are presented in Table S1.

S4. Input parameters to calculations with the time-dependent wave packet method.

Calculations with the time-dependent wave packet (TDWP) method were performed for incidence energies between 0.05 and 1.05 eV. It is not possible to obtain results for this entire energy range in one calculation: it would not be possible to contain the initial wave packet in the appropriate range of momenta directed at the surface and to obtain accurate results at the same time. Therefore, this energy range was split into four smaller energy ranges, and TDWP calculations were performed for these ranges (see Table S4) separately. In each calculation, the *Z*-dependence (*r*-dependence) of the wave function was described with a Fourier grid starting at the start value Z_{start} (r_{start}) provided in Table S4 with N_Z (N_r) subsequent grid points with grid spacings of ΔZ (Δr), as all indicated in Table S4 (the coordinates used have been specified in Fig.1 of the main paper). A projection operator formalism[14] was used to bring in the initial wave packet on a separate, long one-dimensional grid in order to be able to reduce the grid size in Z that is associated with the large scattering basis set. In each calculation this grid has the same start value and grid spacings as the ordinary *Z*-grid, but it has a larger number of points N_Z^{sp} (see Table S4). Furthermore the scattering basis set has periodic Fourier grids in X and Y with N_X and N_Y points, respectively, and in the finite-basis representation (FBR) spherical harmonics are

employed with the maximum value of j equal to j_{max} and the maximum value of m_j equal to m_j^{max} as also given in Table S4.

On the grids in Z and r defined above complex absorbing potentials (CAPS) are defined in Table 4 from a starting value to an end value, and these are stated for the regular Z-grid, the Z-grid used to bring in the initial wave function with the projection operator formalism, and the r-grid using parameters with names that are self-evident. The strength of the squared negative imaginary potentials used as CAPs was taken in such a way that optimal absorption takes place for the translational energy indicated in Table S4 (in eV), using the theory of Ref.[15] and also based on the range of the grid over which the optical potential is defined.

The wave function was propagated with a time step Δt up to a final time t_f selected in such a way that the remaining norm of the wave function on the grid was in all cases less than 0.2%. The initial wave function was centered on the value of Z_0 indicated. Once again these parameters are provided in Table S4.

Table S1. Parameters characterizing the Al(110) slab obtained with the SRP DF in calculations using the static surface approximation and in calculations used to set up a potential energy surface for H_2 + Al(110) for a surface temperature of 220 K. Experimental parameters (in brackets) are taken from the experimental data presented in Ref.[8], and from the analysis performed of these data in Ref.[9]. All interlayer distances are in Å.

Temp (K)	$a_l(\AA)$	$d_{n,n+1}$(bulk)	$d_{1,2}$	$d_{2,3}$	$d_{3,4}$	$d_{4,5}$	$d_{5,6}$
0	4.087<br>(4.027)	1.445<br>(1.424)	1.358<br>(1.3409)	1.489<br>(1.482)	1.399	1.472	1.456
220	4.114<br>(4.054)	1.455<br>(1.434)	1.351<br>(1.334)	1.507<br>(1.500)	1.408	1.483	1.466

Table S2. Results E_{BG1} (in kcal/mol) of DFT convergence tests using the DF with 68% RPBE exchange for the BG1 geometry. Barrier heights computed varying the number of k-points N_k (taken the same in the X and Y directions here), the unit cell size (also taken the same in the X and Y directions here, as N_c x N_c), the smearing energy E_{sm} (in eV) and the order of the Methfessel-Paxton method O_{mp} used with the smearing, the vacuum distance D_V (in Å) and the plane-wave cutoff energy (E_{PW}, in eV) are presented. Finally, N_{ser} labels a series of tests in which one or two parameters were varied while others were kept the same (see the text). The row with numbers in bold face presents the parameters used in the calculations to generate the PES.

N_{ser}	N_k	N_c	E_{sm}	O_{mp}	D_V	E_{PW}	E_{BG1}
1	14	2	0.3	2	24	580	25.18
1	14	3	0.3	2	24	580	25.42
2	8	3	0.3	2	24	460	25.05
2	8	3	0.3	2	24	500	25.18
2	8	3	0.3	2	24	540	25.25
2	8	3	0.3	2	24	620	25.37
2	8	3	0.3	2	24	700	25.40
3	6	3	0.3	2	24	580	25.44
3	8	3	0.3	2	24	580	25.33
3	10	3	0.3	2	24	580	25.46
3	12	3	0.3	2	24	580	25.46
3	14	3	0.3	2	24	580	25.42
4	8	3	0.3	2	12	540	25.62
4	8	3	0.3	2	16	540	25.30
4	8	3	0.3	2	20	540	25.26
4	8	3	0.3	2	24	540	25.25
5	8	3	0.3	1	16	540	25.33
5	8	3	0.2	1	16	540	25.26
**5**	**8**	**3**	**0.1**	**1**	**16**	**540**	**25.22**
5	8	3	0.3	2	16	540	25.30
5	8	3	0.2	2	16	540	25.23
5	8	3	0.1	2	16	540	25.23

Table S3. Parameters characterizing the DFT calculations used to set up the PES for H_2 + Al(110). In the Table, N_{lay} is the number of aluminum layers used in the calculations. The other parameters have been defined in the caption of Table S2.

Parameter	Value	Comments
N_{lay}	10	
N_c	3	
D_V (Å)	16.0	
PAW pseudo-potentials	PAW Al_GW and PAW H_GW	from VASP
N_k	8	Monkhorst-Pack[12]
E_{sm} (eV)	0.1	1st order Methfessel Paxton[3]
E_{PW} (eV)	540.0	

Table S4. Input parameters for the 6D TDWP calculations on the reactive scattering of H_2 from Al(110). The values of j_{max} and of m_j^{max} indicated for $v = 0$ in brackets were the values used in TDWP calculations with initial $j > 11$.

	0.05-0.115 eV		0.10-0.3125 eV		0.3-0.75 eV		0.7-1.05 eV	
	$v=0$	$v=1,2$	$v=0$	$v=1,2$	$v=0$	$v=1,2$	$v=0$	$v=1,2$
$Z_{start}(a_0)$	-1.0	-1.0	-1.0	1.0	-1.0	-1.0	-1.0	-1.0
N_Z^{sp}	512	512	512	512	512	512	512	512
N_Z	180	180	180	180	180	180	180	180
ΔZ (a_0)	0.15	0.15	0.15	0.15	0.15	0.15	0.15	0.15
r_{start} (a_0)	0.4	0.4	0.4	0.4	0.4	0.4	0.4	0.4
N_r	64	64	64	64	64	64	64	64
Δr (a_0)	0.15	0.15	0.15	0.15	0.15	0.15	0.15	0.15
N_x	20	20	20	20	20	20	20	20
N_y	20	20	20	20	20	20	20	20
j_{max}	18 (20)	18	18 (20)	18	18 (20)	18	18 (20)	18
m_j^{max}	12 (14)	12	12 (14)	12	12 (14)	12	12 (14)	12
Z_∞ (a_0)	15.20	15.20	15.20	15.20	15.20	15.20	15.20	15.20
Complex absorbing potential								
Z^{CAP} start [a_0]	15.20	15.20	15.20	15.20	15.20	15.20	15.20	15.20
Z^{CAP} end [a_0]	25.85	25.85	25.85	25.85	25.85	25.85	25.85	25.85
$E(Z^{CAP})$[eV]	0.05	0.05	0.10	0.10	0.25	0.25	0.25	0.25
Z_{sp}^{CAP} start[a_0]	36.80	36.80	36.80	36.80	36.80	36.80	36.80	36.80
Z_{sp}^{CAP}end[a_0]	75.65	75.65	75.65	75.65	75.65	75.65	75.65	75.65
$E(Z_{sp}^{CAP})$ [eV]	0.05	0.05	0.10	0.10	0.25	0.25	0.25	0.25
r^{CAP} start [a_0]	6.85	6.85	6.85	6.85	6.85	6.85	6.85	6.85
r^{CAP} end [a_0]	9.85	9.85	9.85	9.85	9.85	9.85	9.85	9.85
$E(r^{CAP})$[eV]	0.10	0.10	0.10	0.10	0.10	0.10	0.10	0.10
Propagation								
Δt [a.u.t]	1.0	1.0	1.0	1.0	1.0	1.0	1.0	1.0
t_f[a.u.t]	90000	90000	90000	90000	90000	90000	90000	90000
Initial wave packet								
E_{min} [eV]	0.05	0.05	0.1	0.1	0.3	0.3	0.7	0.7
E_{max}[eV]	0.115	0.115	0.3125	0.3125	0.75	0.75	1.05	1.05
Z_0 [a_0]	26.00	26.00	26.00	26.00	26.00	26.00	26.00	26.00

Supporting Figures.

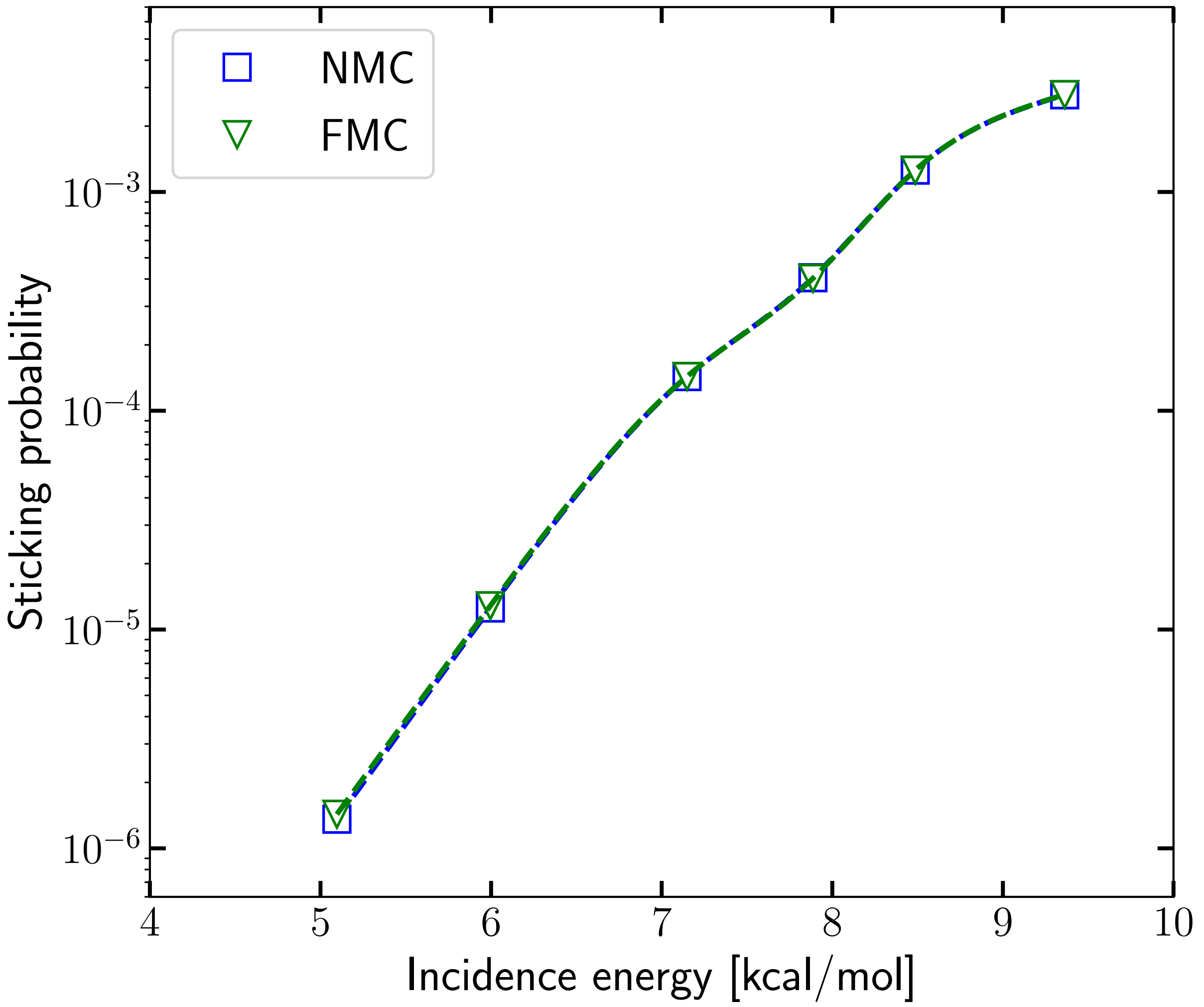

Figure S1. Sticking probabilities computed with the QCT method using the NMC (blue squares) and FMC (green triangles) procedures, as described in the text of the main paper, are compared.

Supporting references.

1. Kresse, G.; Furthmüller, J. Efficient iterative schemes for ab initio total-energy calculations using a plane-wave basis set. *Phys.Rev.B* **1996**, *54*, 11169-11186.
2. Kresse, G.; Joubert, D. From ultrasoft pseudopotentials to the projector augmented-wave method. *Phys.Rev.B* **1999**, *59*, 1758-1775.
3. Methfessel, M.; Paxton, A. T. High-precision sampling for Brillouin-zone integration in metals. *Phys. Rev. B* **1989**, *40*, 3616-3621.
4. Klimes, J.; Bowler, D. R.; Michaelides, A. Van der Waals density functionals applied to solids. *Phys.Rev.B* **2011**, *83*, 195131.
5. Gharaee, L.; Erhart, P.; P. Hyldgaard. Finite-temperature properties of nonmagnetic transition metals: Comparison of the performance of constraint-based semilocal and nonlocal functionals. *Phys.Rev.B* **2017**, *95*, 085147.
6. Granhed, E. J.; Wahnström, G.; Hyldgaard, P. BaZrO3 stability under pressure: The role of nonlocal exchange and correlation. *Phys.Rev.B* **2020**, *101*, 224105.
7. Tran, F.; Stelzl, J.; Koller, D.; Ruh, T.; Blaha, P. Simple way to apply nonlocal van der Waals functionals within all-electron methods. *Phys.Rev.B* **2017**, *96*, 054103.
8. Göbel, H.; von Blanckenhagen, P. Temperature-dependence of interlayer spacings and mean vibrational amplitudes at the Al(110) surface. *Phys. Rev. B* **1993**, *47*, 2378-2388.
9. Powell, A.; Kroes, G. J.; Doblhoff-Dier, K. Quantum Monte Carlo calculations on dissociative chemisorption of H_2 on Al(110): Minimum barrier heights and their comparison to DFT values. *J.Chem.Phys.* **2020**, *153*, 224701.
10. Hammer, B.; Hansen, L. B.; Nørskov, J. K. Improved adsorption energetics within density-functional theory using revised Perdew-Burke-Ernzerhof Functionals. *Phys.Rev.B.* **1999**, *59*, 7413-7421.
11. Perdew, J. P.; Burke, K.; Ernzerhof, M. Generalized gradient approximation made simple. *Phys.Rev.Lett.* **1996**, *77*, 3865-3868.

12. Monkhorst, H. J.; Pack, J. D. Special Points for Brillouin-Zone Integrations. *Phys.Rev.B.* **1976,** *13*, 5188-5192.

13. Berger, H. F.; Rendulic, K. D. An investigation of vibrationally assisted associative desorption: the cases H_2/Cu(110) and H_2/Al(110) *Surf.Sci.* **1991,** *253*, 325-333.

14. Neuhauser, D.; Baer, M. The application of wave-packets to reactive atom—diatom systems - a new approach. *J.Chem.Phys.* **1989,** *91*, 4651-4657.

15. Vibók, Á.; Balint-Kurti, G. G. Parametrization of complex absorbing potentials for time-dependent quantum dynamics calculations. *J. Phys. Chem.* **1992,** *96*, 8712-8719.
